# Ocular cicatricial pemphigoid

**Published:** 2020

**Authors:** Cristina Stan, Emanuela Diaconu, Livia Hopirca, Nicoleta Petra, Anca Rednic, Cristina Stan

**Affiliations:** * University of Medicine and Pharmacy, Cluj-Napoca, Cluj, Romania; ** Hospital of Ophthalmology, Cluj-Napoca, Cluj, Romania; *** Hospital of Pediatric Neurology, Cluj-Napoca, Cluj, Romania

**Keywords:** OCP, dry eye syndrome, entropion, symblepharon

## Abstract

Ocular cicatricial pemphigoid (OCP) is an autoimmune ocular disease that causes severe dry eye syndrome, conjunctival scarring with inferior fornix shortening and entropion along with trichiasis. Corneal keratinization and corneal ulcers may lead to permanent vision loss. The therapeutic approach of OCP is a challenging one. Thus, the treatment consists of a systemic therapy that includes immunosuppressive as well as corticosteroid medication. Also, surgical procedures for modifications of eyelid position, symblepharon and cataract may aggravate the evolution of the disease.

Dry eye syndrome, which is known to be a multifactorial disorder of the ocular surface secondary to qualitative or quantitative alteration of the tear film, is a severe and frequent complication of OCP.

In this article, we presented 3 patients diagnosed with OCP, who developed severe dry eye syndrome, entropion, corneal erosions and ultimately, permanent vision loss.

## Introduction

Ocular cicatricial pemphigoid (OCP) is a form of mucous membrane pemphigoid (MMP) characterized by chronic, relapsing bilateral conjunctivitis. Patients affected by this autoimmune disease will ultimately experience conjunctival cicatrization or scarring and, in case of not responding to treatment, or being left untreated, they will develop corneal opacification and permanent vision loss. MMP affects the skin as well as the mucous membranes of the mouth, nose, esophagus, genitals and anus, leading to erosions, blisters, and strictures. Ocular cicatricial pemphigoid represents about 60-70% of the MMP manifestations.

Ocular cicatricial pemphigoid predominantly affects females twice more than males [**1**], and the age of onset is around 60 years or older. There is no racial predilection. Ocular cicatricial pemphigoid is considered a rare disease and its incidence is estimated to be about 1 per 10,000 to 50,000 [**2**].

Patients with ocular cicatricial pemphigoid experience bilateral chronic conjunctivitis [**3**] with remissions and exacerbations. OCP is often misdiagnosed and delayed due to the insidious onset and nonspecific signs and symptoms in the early stages of the disease [**4**]. In the beginning, patients experience ocular redness, tearing, burning, light sensitivity and foreign body sensation. These presentations are similar to dry eye syndrome and to many other inflammatory conditions of the anterior segment of the eye. There is minimal to no discharge present. As the disease progresses, the signs become pathognomonic for ocular cicatricial pemphigoid, most notably with the development of a symblepharon.

In the early stages of the disease, the conjunctiva exhibits the following signs: diffuse hyperemia, papillary reaction, dry eye syndrome and keratoconjunctivitis sicca due to destruction of goblet cells.

Ocular cicatricial pemphigoid affects the cornea and early manifestations include punctate epithelial erosions, exposure keratitis, epithelial defects, peripheral infiltrates, ulcers and neovascularization. The worsening of the condition results in limbal stem cell failure, which leads to keratinization and conjunctivalization. Corneal opacification is possible in the late stages of the disease. The eyelids are also affected during the process and the first signs of that include blepharitis, trichiasis and entropion, due to subepithelial scarring and keratinization of the eyelid margin [**5**].

## Cases report

**Case 1**

The patient is a 74-year-old female. She was diagnosed with diabetes mellitus. She presented an acute bullous skin eruption after some oral medication 30 years before, of which she had no records. She reported that the eye symptoms, including ocular pain and visual disturbances, began after that episode.

The patient has been in our care for 10 years. Her first visit diagnosis was symblepharon and dry eye syndrome in both eyes with corneal keratinization in the left eye. During the years, she had several acute episodes of corneal ulcer in the right eye (RE), which needed hospitalization, while the left eye (LE) was quiet. The LE had a visual acuity (VA) of light perception and presented an opaque cornea and ankyloblepharon, for which she was treated only with artificial tears. 

The period of time between 2017 and 2018 presented a particular ocular evolution.

Thus, the RE presented several recurrent episodes of corneal erosions, severe corneal neovascularization and severe dry eye symptoms. The treatment consisted in 3 subconjunctival injections of Bevacizumab-Avastin 0,05 ml (1,25 mg), artificial tears and monthly soft contact lens. Meanwhile, she developed cataract in this eye and the visual acuity became “count fingers”. She was told that cataract surgery had poor prognosis. However, she still underwent cataract surgery with posterior chamber IOL implantation. The surgery took place in September 2018. On the other hand, the LE maintained the same biomicroscopic aspect, with no further symptoms or complications.

Nonetheless, in December 2018, 3 months after having the cataract surgery, the RE developed corneal ulcer with aggressive evolution to descemetocele. Local antibiotics, mydriatic and steroids were administrated. No perforation of the cornea happened, instead it healed with a central scar after three weeks.

In the next period, March-April 2019, the RE developed several inflammatory episodes with ocular pain, blurred vision and enlargement of the corneal neovessels. The therapeutic plan included 3 peribulbar injections of 4 mg dexamethasone, one per week. No relief of the pain was observed.

In May 2019, the patient complained of recurrent and short (30 minutes) episodes of acute loss of vision. She was hospitalized because the RE cornea had a new ulcer and was so hazy that the pupil and posterior pole could not be examined. Treatment consisted of antibiotics and dexamethasone in peribulbar injections every 2 days. After one week, some part of the retina could be examined and several retinal hemorrhages were observed. We concluded that it was a retinal venous branch occlusion, but we could not exclude diabetic retinopathy. Neurological and cardiological examinations along with cerebral MRI showed no signs of stroke or arrhythmias. The patient was discharged and the local treatment she continued at home consisted of artificial tears and soft contact lens in both eyes, while the general treatment included Aspenter. VA at that time was “counting fingers at 30 cm” in the RE and light perception in the LE.

**Case 2**

The second patient is a 72-year-old female. She was diagnosed with arterial hypertension. Both eyes presented intense dry eye symptoms for the last two years with no relief from artificial tears.

**Fig. 1:**
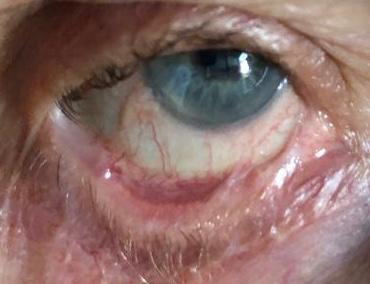
Right eye symblepharon formation

**Fig. 2:**
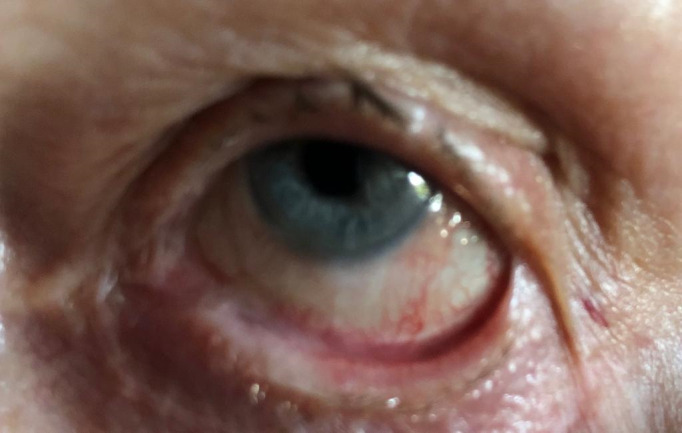
Left eye symblepharon formation

**Fig. 3:**
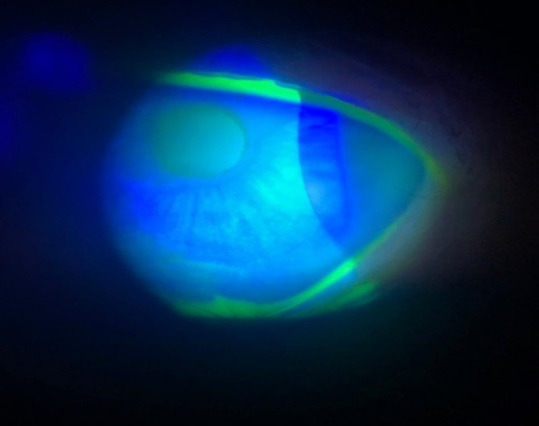
Slit lamp: Corneal punctate epithelial erosions in the right eye

The first visit in our clinic was in May 2018. VA in both eyes was 20/ 20 without correction. Both eyes presented inferior symblepharon, entropion with corneal erosions, and a severe dry eye syndrome. Schirmer 1 test was 0 mm/ 5 min and the tear break up time was 2 sec. No corneal vascularization was observed (**Fig. 1**-**3**).

We started bilateral treatment with local antibiotics, lipid artificial tears during day time and gel during night time, associated with topical steroids for 2 weeks. Because symptoms persisted, she was prescribed topical 1 mg/ ml cyclosporine (Ikervis), 1 drop per day in both eyes. Soon after introducing Ikervis in the therapeutic plan, the patient reported symptoms of ocular pain, hyperemia and blurred vision. Thus, the cyclosporine was discontinued after 3 weeks because the patient did not tolerate it. She continued the local treatment with lipid artificial tears, and in May 2019 she underwent surgery for entropion in both inferior eyelids. A conjunctival biopsy was sampled and examined by direct immunofluorescence, which was positive for IgA, leading to the diagnosis of Ocular cicatricial pemphigoid. The collaboration with a dermatologist led to the patient being immediately treated with immunosuppressive therapy, respectively azathioprine. Systemic oral corticoid treatment was also administered. After 3 months, the patient developed elevated transaminases and the immunosuppressive therapy was interrupted. At present, her treatment consists of lipid artificial tears along with oral corticoid therapy.

**Case 3**

The third patient is 74-year-old male suffering from arterial hypertension. For the last 10 years, both eyes presented dry eye syndrome, corneal neovascularization, symblepharon, misdirection of the cilia and multiple episodes of corneal ulcers, the ulcers being predominant in the right eye. It must be noted that the misdirection of the cilia was present only on the superior right eyelid and the cilia were very soft and did not hurt the corneal epithelium. VA of RE decreased over the years mainly because of the cataract evolution, while VA of LE maintained at “count fingers at 1 meter” due to corneal opacification and neovascularization.

Patient asked intensively for cataract surgery in the RE, despite being strongly recommended not to, considering his pemphigus disease. He had cataract surgery with posterior chamber IOL implantation in March 2016, when his VA was 0,3. After surgery, VA was 0,6 for six months and then began to decrease due to cystoid macular oedema. The patient had 6 intraocular injections with Avastin (1,25 mg), and 4 with Triamcinolone the next year, but VA decreased significantly to “count fingers at 3 meters”. At present, his treatment consists of lipid artificial tears.

## Discussion

***Ocular cicatricial pemphigoid (OCP) is a systemic disease affecting the eye***. The cause of ocular cicatricial pemphigoid is an autoimmune type II hypersensitivity response [**2**]. This autoimmune response occurs when a patient has a genetic predisposition and is exposed to an environmental trigger. Conjunctival biopsy with direct immunofluorescence is the most reliable method and the gold standard to confirm the ocular cicatricial pemphigoid diagnosis.

Ocular complications of ocular cicatricial pemphigoid (OCP) include the following: corneal epithelial defects, corneal stromal ulcers, corneal perforation, endophthalmitis, glaucoma. The differential diagnosis includes all medical conditions that cause an asymmetric bilateral chronic conjunctivitis with conjunctival cicatrization. These include Stevens-Johnson syndrome, toxic epidermal necrolysis, trachoma, graft-versus-host disease, dry eye syndrome, history of adenoviral conjunctivitis, chemical burn, medicamentosa (from topical glaucoma medications and anti-viral medications for herpetic eye disease), atopic keratoconjunctivitis, radiation exposure, systemic lupus erythematosus, and Sjogren syndrome. A distinguishing clinical feature of OCP is a progressive symblepharon [**6**]. Symblepharon from the above etiologies may form and then remain stable. However, a few conditions in which progressive cicatrization occur include neoplasia, lichen planus, and paraneoplastic pemphigus [**6**,**7**].

In the three cases of this study the diagnosis of OCP is sustained by the progressive symblepharon. In case 1 it could have been Steven-Johnson syndrome (30 years ago) but the cicatrization of the conjunctiva progressed aggressively during time. Only case 2 had conjunctival biopsy. Generally, of those with MMP, the ones with ocular involvement have a worse prognosis than the ones affected with skin and/ or oral mucosa involvement alone. Systemic therapy can stop the progression of ocular cicatricial pemphigoid in about 90% of the patients, and the rate of recurrence is about 20% to 30%, but this is variable.

All three cases had only ocular lesions, and case 1 and case 3, with long standing disease, developed corneal opacification and neovascularization. 

OCP is a general disease, therefore, no topical medication can be curative. In selected patients, subconjunctival steroid injections or subconjunctival injections of mitomycin C may be used temporarily to slow disease progression, while systemic therapy takes effect.

Adjuvant treatment with topical lubricants should be used in patients with dry eye symptoms. The use of topical cyclosporine and tacrolimus ointment has also been described to aid in the control of surface inflammation. 

Our patients received topical therapy for dry eye and corneal ulcerations. We did not use general therapy with immunosuppressants because we considered that ocular complications such as corneal ulcers and corneal neovascularization were caused by dry eye syndrome.

Systemic corticosteroids can control the activity of the disease; however, they are not as effective as other immunosuppressive drugs, and the doses required have been shown to be very toxic.

Dapsone is the first-line treatment for mild to moderate disease. Dapsone is a sulfonamide antibiotic that also has anti-inflammatory and immunomodulatory action. It must be avoided in patients with G6PD deficiency due to the risk of hemolytic anemia. Moderate to severe disease or lack of response to Dapsone or other first-line alternatives after 2 to 3 months will likely require a systemic corticosteroid pulse over 6 to 8 weeks with concurrent immunosuppressant therapy with azathioprine, mycophenolate mofetil, methotrexate, or cyclosporine. Cyclophosphamide should be considered in severe and rapidly progressing disease states, especially when previous therapies have been unsuccessful. 

Biologics such as etanercept or rituximab and intravenous immunoglobulin therapy are reserved for patients who have a poor response to conventional therapy.

The need for cataract surgery is common in patients with OCP. Cataract surgery performed on patients with OCP is followed by increased conjunctival inflammation, rapid progression of keratopathy, and conjunctival scarring if the disease is not medically controlled.

Two of our patients (case 1 and case 3) underwent cataract surgery and both had a favorable course for six months postoperatively and ocular complications occurred after that period. None of them presented progression of symblepharon years before and after the surgery, but active disease could not be ruled out.

Corneal transplantation on a dry eye with impaired lid function and limbal stem cell deficiency has a very poor prognosis; therefore, corneal grafting in patients with advanced OCP should be avoided. This procedure should only be performed in case of corneal perforation. In patients with advanced corneal damage from OCP keratoprosthesis may be the only feasible alternative for visual rehabilitation.

Entropion surgery is usually avoided in patients with OCP because of the interference with the conjunctiva. Several cases of lower lid entropion have been treated successfully with a retractor plication technique. The procedure is repeatable in case of undercorrection. Moreover, the conjunctiva remains intact during the surgery, which can avoid the exacerbation of conjunctival inflammation.

In case 2, the entropion surgery consisted of resection of a lamellae of lower palpebral skin (parallel with the entire eyelid margin and 9 mm width) in order to avoid hurting the conjunctiva. In this case, the treatment with methotrexate would be considered because of the progressive symblepharon, while the symptoms of dry eye syndrome are well controlled with artificial tears.

## Conclusions

Ocular cicatricial pemphigoid is a general autoimmune disease with ocular manifestations that could lead to blindness. Ocular complications are dry eye, corneal ulcers, corneal neovascularization and entropion. Even if the disease is inactive due to natural evolution or to treatment effect, the ocular sequelae threatens the visual function and the patient must be supervised all his/ her life.

The pathognomonic sign of OCP is progressive symblepharon. 

The disease must be treated by a multidisciplinary approach. Thus, the treatment must be systemic and needs to be concluded by a medical team composed of ophthalmologists, rheumatologists, dermatologists.
